# Phage endolysins as new therapeutic options for multidrug resistant *Staphylococcus aureus*: an emerging antibiotic-free way to combat drug resistant infections

**DOI:** 10.3389/fcimb.2024.1397935

**Published:** 2024-06-17

**Authors:** Melaku Ashagrie Belete, Selamyhun Tadesse, Mihret Tilahun, Ermiyas Alemayehu, Muthupandian Saravanan

**Affiliations:** ^1^ Department of Medical Laboratory Science, College of Medicine and Health Sciences, Wollo University, Dessie, Ethiopia; ^2^ Department of Medical Laboratory Science, College of Health Sciences, Woldia University, Woldia, Ethiopia; ^3^ Department of Pharmacology, AMR and Nanotherapeutics Laboratory, Saveetha Dental College and Hospitals, Saveetha Institute of Medical and Technical Sciences (SIMATS), Chennai, Tamil Nadu, India

**Keywords:** phage endolysins, new therapeutic options, multidrug resistant *Staphylococcus aureus*, MDR - *S. aureus*, combating AMR

*Staphylococcus aureus* (*S. aureus*) has developed various drug resistance mechanisms, which make it difficult to treat with conventional antibiotics. The most common drug resistance mechanisms of *S. aureus* include: productions of beta-lactamase, an enzyme that breaks down beta-lactam antibiotics such as penicillins and cephalosporins; methicillin resistance [methicillin-resistant *S. aureus* (MRSA)], a genetic mutation that allows them to produce an altered penicillin-binding protein (PBP2a), which has a reduced affinity for methicillin and other beta-lactam antibiotics; vancomycin resistance [vancomycin-resistant *S. aureus* (VRSA)], acquired ability to modify their cell wall structure, which reduces the effectiveness of vancomycin; macrolide resistance; aminoglycoside resistance; quinolone resistance, and biofilm formation (Hiramatsu et al., 2014).

Nowadays, recent scientific advances confirmed a novel antibiotic-free means to combat *S. aureus* antimicrobial resistance. This new therapeutic option is the use of endolysins, enzymes produced by bacteriophages, that can hydrolyze the peptidoglycan layer of bacterial cell walls, leading to bacterial lysis and death. Several studies have demonstrated the efficacy of endolysins in killing multidrug resistant *S. aureus* such as MRSA and VRSA *in vitro* and *in vivo*, including those that are resistant to conventional antibiotics (Schmelcher et al., 2012; Mishra et al., 2021).

Endolysins are composed of one N-terminal enzymatically active domain (EAD) and one C-terminal cell wall-binding domain (CBD), connected by a short linker region. Endolysins work by binding to the peptidoglycan layer of the bacterial cell wall and cleaving the bond between the N-acetylmuramic acid (NAM) and N-acetylglucosamine (NAG) subunits, unique structures not present in mammalian cells, through specific mechanisms including N-acetylmuraminidase activity to cleave the glycosidic bond between NAM and NAG resembling the action of lysozymes, and endopeptidase activity to cleave the peptide cross-links between amino acid residues in the peptidoglycan layer, resembling the action of endopeptidases (Rahman et al., 2021). Additionally, endolysins exert N-acetylglucosaminidase activity involving hydrolysis of the β-1,4-glycosidic bond between NAG residues in the peptidoglycan layer, transglycosylase activity involving the cleavage of glycosidic bond between sugar residues of peptidoglycan layer facilitating insertion of new peptidoglycan units, lipoteichoic acid hydrolase activity involving hydrolysis of the ester bonds within lipoteichoic acid, and amidase activity involving hydrolysis of the amide bond between NAM and L-alanine residues in the peptidoglycan layer (Schmelcher et al., 2012; Rahman et al., 2021). These enzymatic activities lead to the breakdown of the cell wall and subsequent lysis of the bacterial cell. This means that endolysins are highly specific for their target bacteria and do not affect mammalian host cells, have a low risk of toxicity to human cells, making them a potentially safer alternative to conventional antibiotics (Rahman et al., 2021).

Several studies have demonstrated the efficacy of endolysins in killing MRSA both *in vitro* and *in vivo*. For instance, a recent study found that a chimeric endolysin composed of lysostaphin and lysostaphin-like domain (LysKLD) was effective against 23 out of 24 clinical MRSA isolates, including those that were resistant to conventional antibiotics (Yang et al., 2019). In a comparable study, Lu Yifei demonstrated a robust antimicrobial activity of a chimeric endolysin LysP108 against *S. aureus* including MRSA *in vitro* (Lu et al., 2021). Similar antibacterial activity of a chimeric endolysin Lys109 against MRSA was reported (Son et al., 2021). Another study showed that a combination of endolysin and vancomycin was more effective in treating MRSA and VRSA infections in mouse models than vancomycin alone. In addition, engineered endolysins LysECD7-SMAP and MR10, belonging to the class of enzybiotics, have been shown to have synergistic effects when combined with conventional antibiotics (Chopra et al., 2016; Arshinov et al., 2022). Recent advances further proved the improved efficacy of endolysins LysSP1 and LysPN09, with the presence of EDTA (Jiang et al., 2021; Ni et al., 2021). The incorporation of edible ϵ-poly-L-lysine (EPL), and weak organic acids including citric acid and malic acid were demonstrated to enhance the antibacterial activity of endolysins (Oliveira et al., 2016; Han et al., 2019). A novel endolysin XZ.700 were also found to be effective in treating MRSA biofilms without showing toxicity on human bone cells *in vitro* (Kuiper et al., 2021). On the other hand, researchers have recently focused on the development of nanotechnology-based delivery vehicles for phage endolysins which allow the delivery of endolysins to infection sites boosting their efficacy (Kashani et al., 2018). Experimental evidence was provided regarding the inhibitory effect of recombinant endolysin XZ.700 against *S. aureus* skin colonization and malignant T cell activation in the case of cutaneous T cell lymphomas (CTCL) blocking *S. aureus* induction of Interferon-gamma (IFNγ) and IFNγ-inducible chemokine CXCL10 in mice skin and proliferation of pathogenic *S. aureus* (Pallesen et al., 2023). An *in vivo* and *in vitro* study further revealed the efficacy of an engineered phage endolysin LysRODAmi and ClyRODI-H5 in removing preformed biofilm, preventing new biofilm formation, and killing drug resistant *S. aureus* in both intact and disrupted keratinocyte monolayers without toxicity toward human keratinocytes (Gutiérrez et al., 2021).

Despite their promise, there are few challenges associated with the use of endolysins as therapeutics, including the potential for development of resistance to endolysins over time probably due to an altered cell wall structures capable of avoiding recognition by endolysins, the difficulty of delivering endolysins to the site of infection as endolysins can be rapidly cleared by the immune system, and too expensive production costs, which could limit their widespread availability. Endolysins are generally promising therapeutic option for the treatment of multidrug resistant *S. aureus* including MRSA, and other bacterial infections. Their specificity and low risk of toxicity make them potentially safer alternatives to conventional antibiotics. Strategies such as combination therapy with antibiotics or other substances, and nanotechnology-based delivery systems are demonstrated to overcome the limitations associated with their use. However, further research is needed to fully understand their potential as therapeutics and to address the challenges associated with their use.

## Author contributions

MB: Writing – review & editing, Writing – original draft, Visualization, Validation, Supervision, Software, Resources, Project administration, Methodology, Investigation, Funding acquisition, Formal analysis, Data curation, Conceptualization. ST: Writing – review & editing, Visualization, Supervision, Investigation, Data curation. MT: Writing – review & editing, Visualization, Validation, Supervision, Investigation. EA: Writing – review & editing, Visualization, Validation, Supervision, Investigation. MS: Writing – review & editing, Visualization, Validation, Supervision, Project administration, Investigation, Conceptualization.
